# Conservation and threatened status of plant species with extremely small populations in the karst region of southeastern Yunnan, China

**DOI:** 10.3389/fpls.2024.1520363

**Published:** 2024-12-24

**Authors:** Yang Liu, Yu-Lin Tan, Yun-Meng Li, Yan-Mei Ping, De-Ming He, Gui-Liang Zhang, Wei-Bang Sun, Lei Cai

**Affiliations:** ^1^ Yunnan Key Laboratory for Integrative Conservation of Plant Species with Extremely Small Populations, Kunming Institute of Botany, Chinese Academy of Sciences, Kunming, Yunnan, China; ^2^ State Key Laboratory of Plant Diversity and Specialty Crops, Kunming Institute of Botany, Chinese Academy of Sciences, Kunming, Yunnan, China; ^3^ University of Chinese Academy of Sciences, Beijing, China; ^4^ Kunming Botanical Garden, Kunming Institute of Botany, Chinese Academy of Sciences, Kunming, Yunnan, China; ^5^ Forestry and Grassland Bureau of Hekou Yao Autonomous County, Hekou, Yunnan, China; ^6^ Wenshan National Nature Reserve Administration, Wenshan, Yunnan, China; ^7^ Hekou Branch Administration of Daweishan National Nature Reserve, Hekou, Yunnan, China

**Keywords:** southeastern Yunnan, conservation status, threatened status, PSESP, karst region

## Abstract

The southeastern Yunnan is one of the most typical areas in China with karst landforms. The rich variety of vegetation types and plant diversity means that threatened status are also synchronized. Over the past 20 years, the comprehensive conservation team for plant species with extremely small populations (PSESP) has conducted in-depth field surveys in the region, combining relevant literature and conservation projects to compile a list of PSESP which including conservation and endangered status, conservation actions, and scientific research. Among all 116 PSESP, relatively abundant families include Cycadaceae (12 species), Magnoliaceae (17species) and Orchidaceae (18 species). Hekou and Malipo are the counties with the highest number, with 44 and 43 species respectively. A total of 81 species are included in the *List of National Key Protected Wild Plants in China*. For threatened status, 24 critically endangered (CR) species and 41 endangered (EN) species represent levels of severe threat. Up to now, 96 species have taken at least one protective measure from *in situ* conservation, ex situ conservation, breeding or reintroduction/reinforcement. But there are still 20 species that have not taken any protective measures. Additionally, scientific research has been conducted on 86 species, but 30 species have not had any research initiated. The threat of human interference mainly including overcollection and habitat destruction, and the threats of limitations imposed on PSESP itself and natural disasters cannot be ignored. Our findings underscore the importance of integrated conservation strategies, in addition to the *in situ* conservation, ex situ conservation, breeding or reintroduction/reinforcement, we should also pay attention to the scientific research, germplasm conservation, environmental education and ethnic culture. We also propose to consider establishing a professional karst botanical garden in southeastern Yunnan, and hope this study can offer valuable insights for the conservation of PSESP and biodiversity in southeastern Yunnan.

## Introduction

Biodiversity is the foundation of human social development and survival (Jiang and Ma, 2014; Wei et al., 2021). Thus, biodiversity conservation, especially for severely threatened species in hotspot areas, has always been a hot topic (Alpert, 1996; Berry et al., 2018; Volis, 2018; Mi et al., 2021; Qin et al., 2023; Cai et al., 2024a). Meanwhile, biodiversity has also been severely impacted by rapid changes in global climate and environment, as well as interference from human activities (Fischer et al., 2013; Tittensor et al., 2014; Senior et al., 2024). In contrast, threatened species face higher risk of extinction compared to other species (Sun et al., 2019a, 2019b, 2021; Senior et al., 2024). In recent years, for some species that require priority rescue and protection, the concept of plant species with extremely small populations (PSESP) has been proposed in Yunnan, China, which have four distinct characteristics: small population size, narrow or fragmented habitats, severe human disturbance, and the risk of imminent extinction (Ma et al., 2013; Sun, 2013; Sun et al., 2019b). After years of development, this new concept of conservation biology has been recognized by many scholars and government decision-makers, as reflected in related scientific research and project support (Sun et al., 2019a; Crane, 2020; Yang et al., 2020; Cogoni et al., 2021; Gratzfeld et al., 2022; Sun et al., 2024).

Southeast Yunnan is located in the border area between China and Vietnam, adjacent to the Indochinese Peninsula, and belongs to the northern edge of tropical rainforests (Shui and Chen, 2006; Cai, 2020). It is also one of the important regions of China’s flora with extremely rich plant diversity, especially with numerous endemic and endangered plant species (Myers et al., 2000; Tian, 2014; Tian et al., 2015; Cai, 2020). The region features a diverse range of karst landforms, including typical karst peak forests, basins, hills, and deeply incised “V”-shaped karst canyons. The altitude in southeastern Yunnan ranges from the lowest point (76.4 m) at the confluence of the Nanxi River and the Red River to the highest point (3,074 m) at the peak of Xilong Mountain (Shui and Chen, 2006). The diverse terrain and landforms, huge altitude differences, warm climate, and abundant rainfall in the karst areas of southeastern Yunnan have created the characteristics of diverse vegetation types, rich plant diversity, diverse endemic groups, and ancient origins of plant flora (Shui, 2003; Shui and Chen, 2010; Cai, 2020). The vegetation types transition from low to high altitudes as follows: tropical rainforests and monsoon evergreen broad-leaved forests—monsoon evergreen broad-leaved forests—mountain moss evergreen broad-leaved forests—summit moss dwarf forests (Zhu, 2023). As of today, there are over 8,000 species of higher plants in the southeastern region of Yunnan.

There are many ancient relict groups distributed in karst region in southeastern Yunnan, which are also shelters for many PSESP studies that have been conducted, such as Magnoliaceae spp., *Cycas* spp., *Camellia* spp. and *Craigia yunnanensis* (Chen, 2017; Guan, 2013; Chen, 2020; Yang, 2022; Liu, 2024). Cai conducted a study on the conservation status, threatened factors, and potential distribution areas of 80 PSESP species distributed in southeastern Yunnan in 2020, and also proposed corresponding protection measures (Cai, 2020). Research and government departments have carried out protection and research on many PSESPs distributed in southeastern Yunnan, but there is still a lack of comprehensive research on their conservation and threatened status at present. The unique ecological and geographical habitats of the region mean that most species have small population sizes, narrow natural geographical distributions, low adaptability, and are highly sensitive to external disturbances. They are extremely vulnerable to becoming endangered or even extinct after natural disasters and human destruction, making the significance of conducting research on PSESPs in this area self-evident (Cai, 2020).

Through field investigations, it was found that relevant forestry and nature reserve departments in the jurisdiction have carried out some simple surveys and protection work on PSESP. This means that the specific quantities of PSESP resources in each county/city are unclear, the protection status is not optimistic, and the research and protection work are insufficient. Many PSESP still face the danger of imminent extinction (Sun et al., 2019a). Since the concept of PSESP was proposed, corresponding protection policies and lists have been introduced at the national and provincial levels (Ma et al., 2013; Sun et al., 2019a; Sun, 2021). However, the diversity, distribution patterns and threatened factors of PSESP in the southeastern region of Yunnan are still not clear. This study comprehensively counts and summarizes the latest status of diversity, distribution, endangered, threatened factors, conservation action and scientific research for PSESP distributed in Southeast Yunnan. By statistically analyzing these data, the aim is to identify distribution patterns and provide theoretical support for further protection efforts.

## Materials and methods

### Study area and climatic condition

The Southeast Yunnan region referred to in this study is a traditional geographical unit in an administrative sense. If according to the division method in the flora map of Yunnan Province, it not only includes the Southeast Yunnan plant region but also parts of the China-Vietnam border region and the China-Myanmar-Laos border region. In terms of plant flora, it mainly includes components of tropical and subtropical plant regions (Zhu, 2023). Geographically, Southeast Yunnan mainly includes the Honghe Hani and Yi Autonomous Prefecture (Honghe) and the Wenshan Zhuang Autonomous Prefecture (Wenshan), covering an area of 65,170 km² with a total of 21 counties and cities. The majority of Honghe Prefecture belongs to the subtropical plateau monsoon climate, with an average annual temperature of 15–22.6°C and an average annual precipitation of 810–2,280 mm. Wenshan Prefecture is located in the southeastern part of the Yunnan-Guizhou Plateau and has a subtropical humid monsoon climate, with an average annual temperature of 12.0–23.1°C. There is a clear distinction between the dry and wet seasons, with May to October being the rainy season, accounting for 82% of the annual rainfall; November to April of the following year is the dry season, accounting for 18% of the annual rainfall (http://www.weather.com.cn). There are 13 nature reserves in southeastern Yunnan, which are four national nature reserves: Daweishan, Jinping Fenshuidiling, Lvchun Huanglianshan, and Wenshan, as well as nine provincial nature reserves: Armushan in Honghe, Guanyinshan in Yuanyang, Yanzidong in Jianshui, Gulinqing in Maguan, Laojunshan in Malipo and Maguan, Laoshan in Malipo, Babao in Guangnan, Tuoniangjiang in Funing, and Puzhehei in Qiubei in Southeast Yunnan. We will conduct statistical analysis on all PSESPs in the southeastern Yunnan region.

### Data sources and statistical analysis

The list of PSESP in Southeast Yunnan was mainly compiled based on the following related protection lists: 62 species in the “Yunnan Province Plant Species with Extremely Small Populations Rescue and Conservation Plan Outline (2010–2020) and Emergency Action Plan (2010–2015)” approved by the People’s Government of Yunnan Province in 2010; 120 species prioritized for protection by the end of 2015 proposed in the “National Plant Species with Extremely Small Populations Rescue and Conservation Project Plan” (2010–2015) jointly issued by the State Forestry Administration and the National Development and Reform Commission in 2012; 231 species stipulated in the project “Survey and Germplasm Conservation of Plant Species with Extremely Small Populations in Southwest China” (2017FY100100) by the Ministry of Science and Technology; 110 species in the “List of Yunnan Protected Plant Species with Extremely Small Populations (2021); 50 species proposed in the “14th Five-Year” forestry and grassland protection development plan in 2021; and 100 species in the “14th Five-Year” national Plant Species with Extremely Small Populations rescue and conservation construction plan in 2022. By organizing and summarizing these lists, a list of PSESP located in Southeast Yunnan is compiled.

By using Excel to organize the data of PSESP obtained from statistics. Based on the organized results, use Chiplot to create bar charts and Sankey diagrams (https://www.chiplot.online/), and use jvenn to draw Venn diagrams (Philippe et al., 2014).

### Taxonomic system and distribution

The selected PSESP mainly include three categories: pteridophytes, gymnosperms, and angiosperms. The classification system selected for the database includes the PPG I system (Schuettpelz et al., 2016), Yang Yong’s system (Yang et al., 2022a), and the APG IV system (Byng et al., 2016), respectively. Based on the results of field investigations, combined with academic monographs such as “*Flora Yunnanica*” (Wu, 1995) and “Seed plants of the karst region in China” (Shui and Chen, 2006), specimen records, nature reserve survey reports, and relevant published academic papers, the distribution areas of each PSESP are determined. Geographic distribution data is collected and organized to establish a geographic distribution database. ArcGIS 10.8 is used in conjunction with species distribution and the geographical location maps of the 21 counties and cities in Southeast Yunnan to draw species distribution maps, and the richness of PSESP in different counties and cities is statistically analyzed by Excel software.

### Conservation level and conservation action

The conservation level of the selected PSESP was determined based on the latest *List of National Key Protected Wild Plants in China* (2021 edition) published by National Forestry and Grassland Administration (www.forestry.gov.cn/main/3457/20210915/143259505655181.html). The current conservation strategies mainly include the following four methods: (1) *In situ* conservation, which involves establishing nature reserve or conservation sites within the native habitats of PSESP to protect existing individuals and their habitats; (2) Ex situ conservation, a method that involves moving plants from their natural habitats to botanical gardens, nurseries, or other locations outside their natural habitats for artificial care (Sun, 2024); (3) Breeding, which is the process of reproducing plants through artificial or natural methods, such as seeds, seedlings, rhizomes, and branches, to cultivate new plant individuals; (4) Reintroduction/Reinforcement, a form of protection that involves selecting suitable sites within the historical or current distribution range to plant artificially propagated individuals, aiming to rebuild or restore natural populations, which also includes enhanced reintroduction, i.e., expanding population size or changing population structure (Sun, 2013; Sun et al., 2019a). The conservation status of the species is recorded mainly through field investigations, and coupled with literature review, and conservation collaborative projects (Sun et al., 2019a; Cai, 2020; Yang et al., 2020; Sun, 2021).

### Threatened level and threatened factors

The threatened levels were recorded mainly by *The China Biodiversity Red List Higher Plants Volume* (2020) and comparing the IUCN Red List Categories and Criteria (Version 2024-1). The threatened levels mainly include the following six classes: Critically Endangered (CR); Endangered (EN); Vulnerable (VU); Near Threatened (NT); Least Concern (LC); Data Deficient (DD) (Qin et al., 2017; Sun, 2021). Through field investigations and studies of PSESP distributed in Southeast Yunnan, it is found that the threatened factors of species are diverse and comprehensive. However, they can be summarized into the following three main aspects: human activity interference, self-factor limitations, and climate change and natural disasters (Cai, 2020; Su et al., 2024).

### Scientific research

Conducting scientific research related to the conservation biology of PSESP is an essential foundation for comprehensive conservation efforts. By reviewing Chinese literature (China National Knowledge Infrastructure, https://www.cnki.net/), English literature (Web of Science, https://webofscience.clarivate.cn), dissertations (Dissertation Knowledge Discovery System) and other materials. The research status of PSESP in Southeast Yunnan is documented in the following four aspects: population surveys, reproductive biology, conservation genetics, and soil microbiology studies.

## Results

### Species diversity and composition

**Figure 1:**
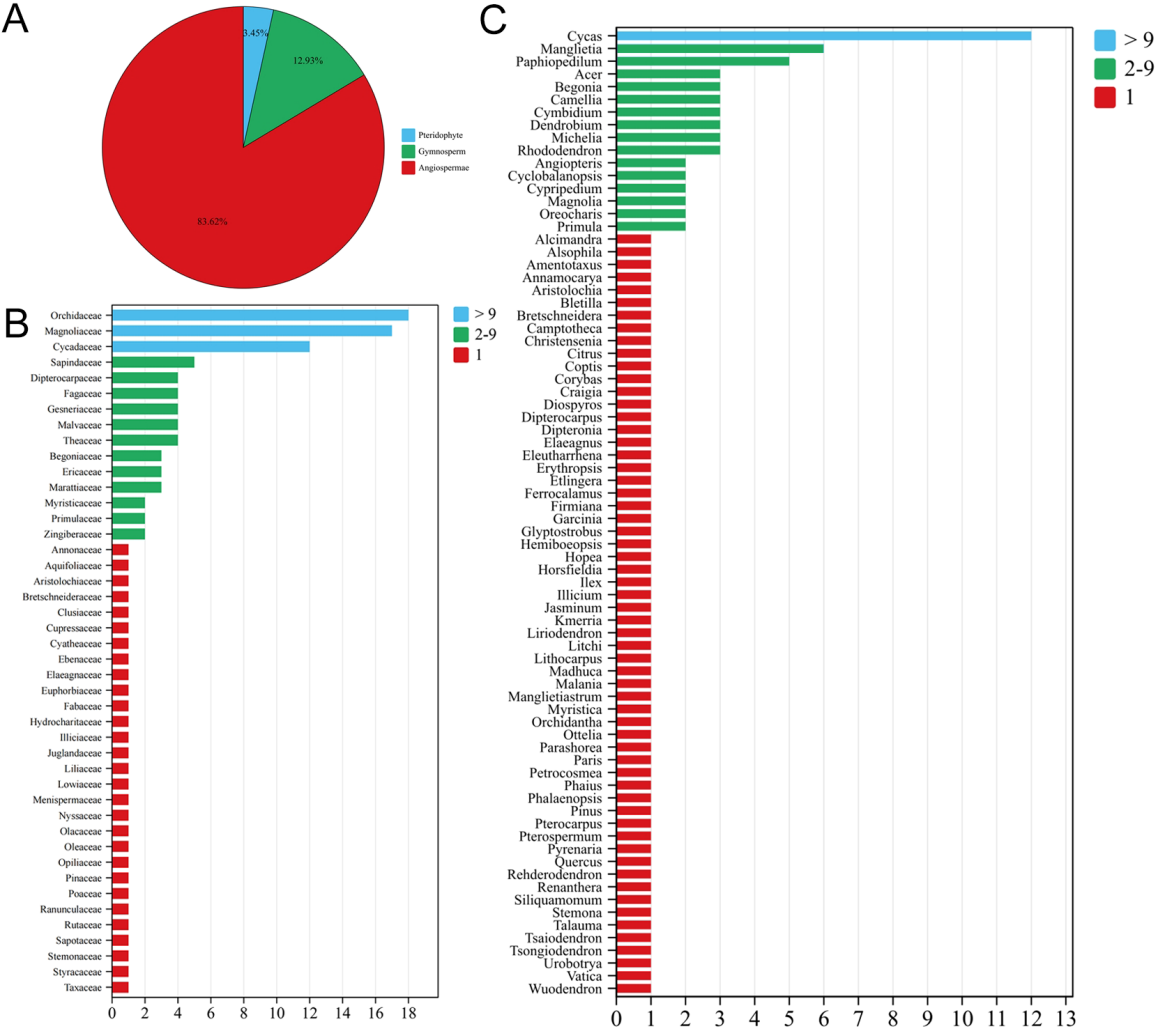
**(A)** Percentages of pteridophytes, gymnosperms, and angiosperms in the list, **(B)** number of species in the different families, **(C)** number of species in the different genera.

Based on the aforementioned related lists, a total of 116 PSESP were identified in Southeast Yunnan, belonging to 44 families and 76 genera (**Supplementary Table S1**). Among them, pteridophytes, gymnosperms, and angiosperms account for 4 species (3.45%), 15 species (12.93%), and 97 species (83.62%), respectively (**Figure 1A**). The proportion of PSESP in the Orchidaceae, Magnoliaceae, and Cycadaceae families is the highest, with 18 species (15.52%), 17 species (14.66%), and 12 species (10.34%), respectively. At the genus level, the three genera with the highest proportion are *Cycas* with 12 species (10.34%), *Manglietia* with 6 species (5.17%), and *Paphiopedilum* with 5 species (4.31%).

According to the number of PSESP contained in each family, there are up to 29 families with one species listed in the directory, accounting for 25% of the total number of species. The families with more than ten species are Orchidaceae, Magnoliaceae, and Cycadaceae, with a cumulative total of 47 species, accounting for 40.52%. There are 12 families with 2–5 species, namely Sapindaceae (5), Dipterocarpaceae (4), Fagaceae (4), Gesneriaceae (4), Malvaceae (4), Theaceae (4), Begoniaceae (3), Ericaceae (3), Marattiaceae (3), Myristicaceae (2), Primulaceae (2), and Zingiberaceae (2), with a cumulative total of 40 species, accounting for 34.48%. (**Figure 1B**). Similarly, based on the number of PSESP contained in each genus, the genus *Cycas* is exceptional, with over ten species, accounting for 12 species (10.34%). There are 15 genera with 2–6 species, accounting for 19.74% of all genera, with 44 species, accounting for 37.93% of all species. There are 60 genera with only one species, accounting for 78.95% of all genera, with 60 species, accounting for 51.73% of all species (**Figure 1C**).

### Species richness and distribution

**Figure 2:**
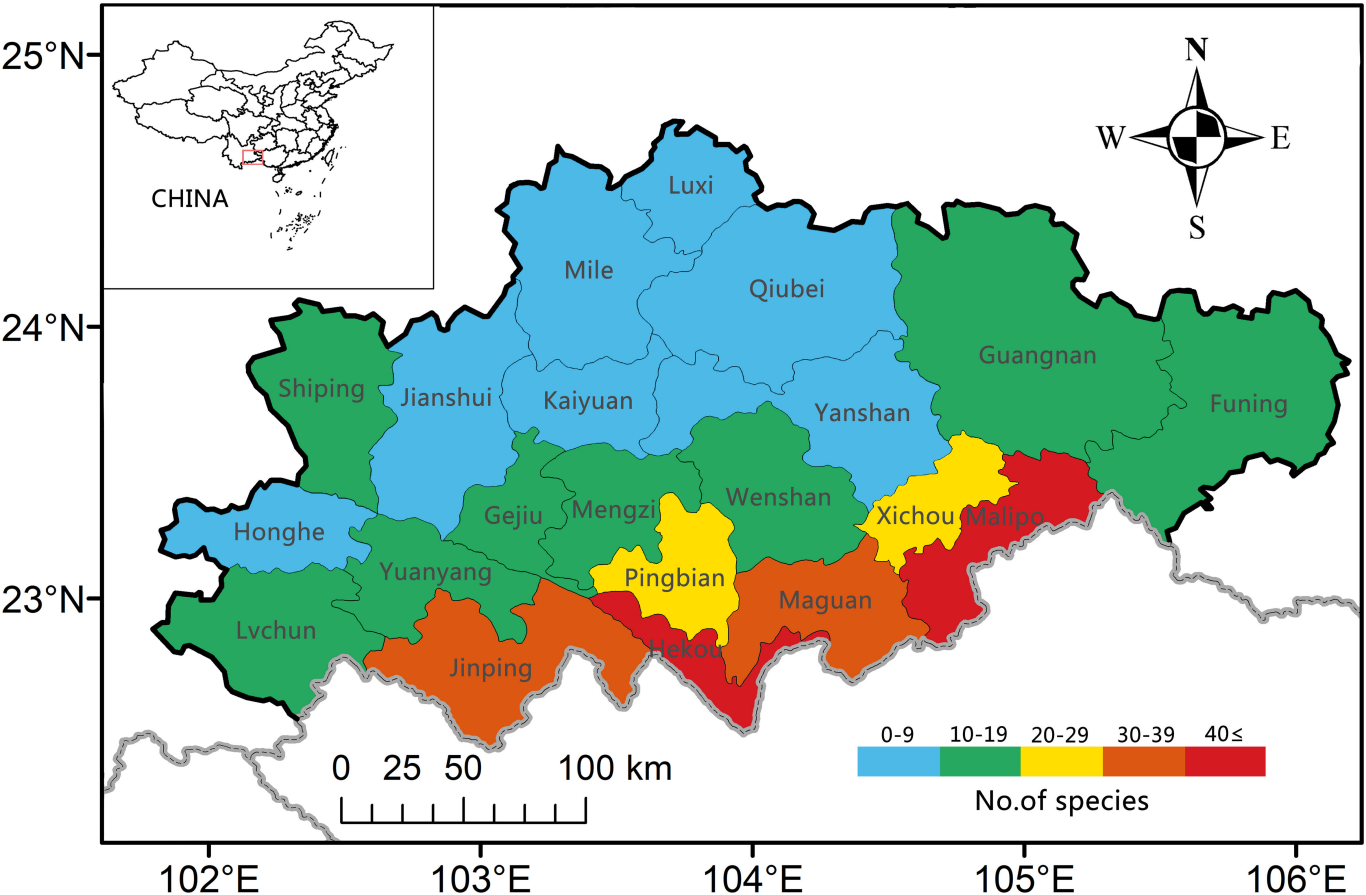
Geographical distribution and the number of PSESPs distributed in various counties and cities in southeastern Yunnan.

By combining the results of field investigations, literature records, and herbarium specimens, the species richness of PSESP distributed across 21 counties and cities in Southeast Yunnan was statistically counted and ranked (**Supplementary Table S2**). The current status of species numbers in each county/city can be divided into five grades: 0–9 species, 10–19 species, 20–29 species, 30–39 species, and 40 species or more. Among them, there are seven counties/cities with 0–9 species, including Yanshan (7), Jianshui (5), Honghe (4), Mile (2), Qiubei (2), Kaiyuan (1), and Luxi (1); there are eight counties/cities with 10–19 species, which are Lvchun (19), Gejiu (18), Wenshan (18), Funing (16), Mengzi (12), Shiping (12), Yuanyang (11), and Guangnan (10); the regions with 20–29 species are two, Pingbian (27) and Xichou (20); the regions with 30–39 species include Maguan (35) and Jinping (31); the regions with 40 species or more include Hekou (44) and Malipo (43) (**Figure 2**; **Supplementary Table S2**).

### Conservation level and threatened level analysis

The conservation level for the 116 PSESP was divided into three levels: (I) National First-Class Key Protected Wild Plants (28 species accounting for 24.14%) and (II) National Second-Class Key Protected Wild Plants (53 species accounting for 45.69%) were protected the national government, and others (35 species accounting for 30.17%) are unprotected (**Supplementary Table S2**). The species at class I mainly belong to the families Cycadaceae (12), Orchidaceae (7), Dipterocarpaceae (3), Magnoliaceae (2). The species at class II mainly belong to the families Orchidaceae (10) and Magnoliaceae (7).

**Figure 3:**
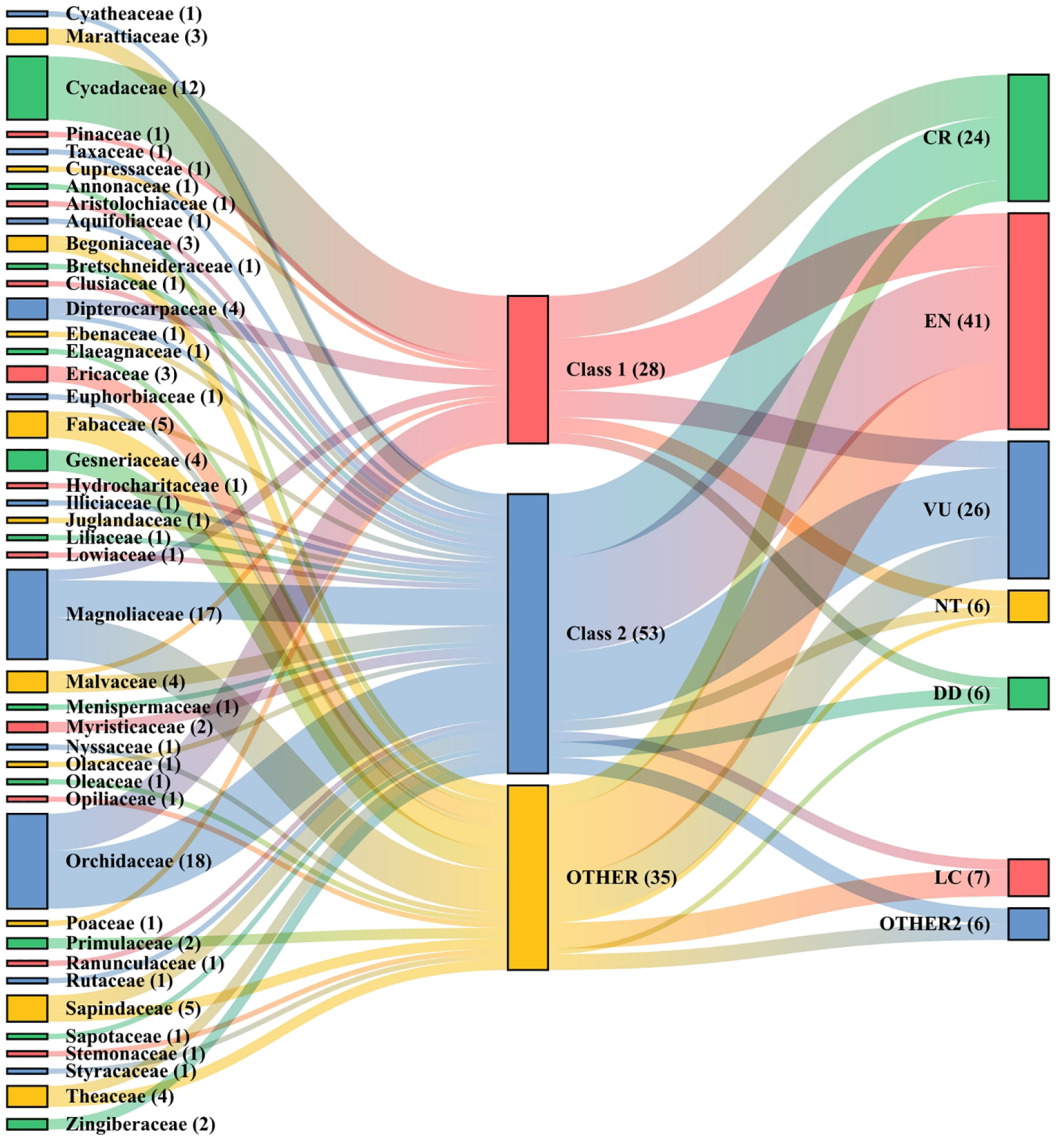
National conservation levels and threatened levels of different species of the listed families.

Based on the combination of the *China Biodiversity Red List Higher Plants Volume* (2020) and the IUCN Red List, the threatened levels of the selected PSESP are recorded (**Supplementary Table S2**). Among them, there are 24 species at the CR level (20.69%), 41 species at the EN level (35.34%), 26 species at the VU level (22.41%), 6 species at the NT level (5.17%), and 6 species with DD (5.17%) (**Figure 3**).

### Conservation action

**Figure 4:**
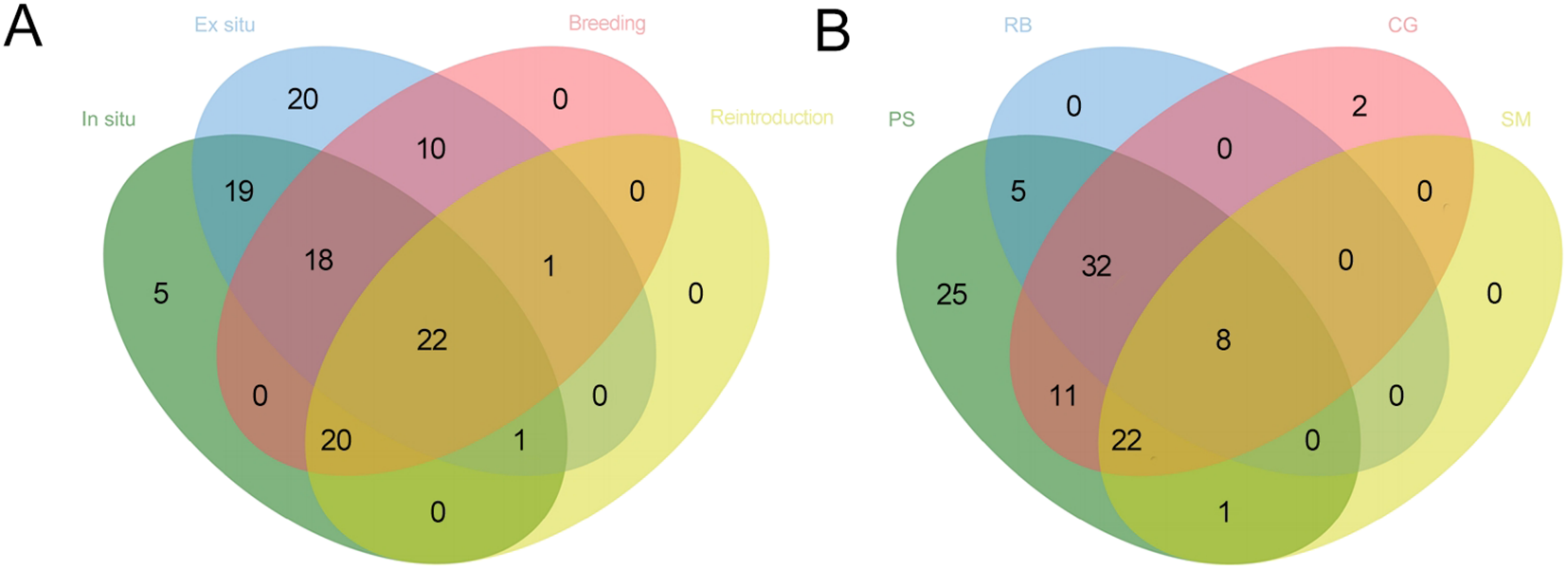
**(A)** Conservation measures implemented for the species, **(B)** Scientific studies that have been conducted on the species (PS, Population Survey; RB, Reproductive Biology; CG, Conservation Genetics; SM, Soil Microorganism).

Based on the survey of the conservation actions of PSESP in Southeast Yunan, there are 65 species undergoing *in situ* conservation, accounting for 56.03%; 91 species undergoing ex situ conservation, accounting for 78.45%; 51 species undergoing breeding, accounting for 43.97%; and 24 species undergoing reintroduction/reinforcement, accounting for 20.69%. Among them, 25 species have only one conservation method implemented, 29 species have two conservation measures taken, 20 species have three conservation measures taken, and 22 species have all four conservation methods implemented. However, there are still 20 species that have not had any conservation measures taken to date, and future conservation efforts should focus on implementing appropriate conservation methods for these species (**Figure 4A**).

### Scientific research

Through literature review, we have recorded the four main research directions for the 116 species. There are 84 species that have undergone population survey research, accounting for 72.41% of the total; 45 species have had reproductive biology research conducted, accounting for 38.79%; 56 species have had conservation genetic research conducted, accounting for 48.28%; and 11 species have had soil microbial research in their habitats, accounting for 9.48%. Among them, 59 species have had two or more research projects conducted, however, there are still 30 species that have not had any research conducted (**Figure 4B**).

### Threatened factors

**Figure 5:**
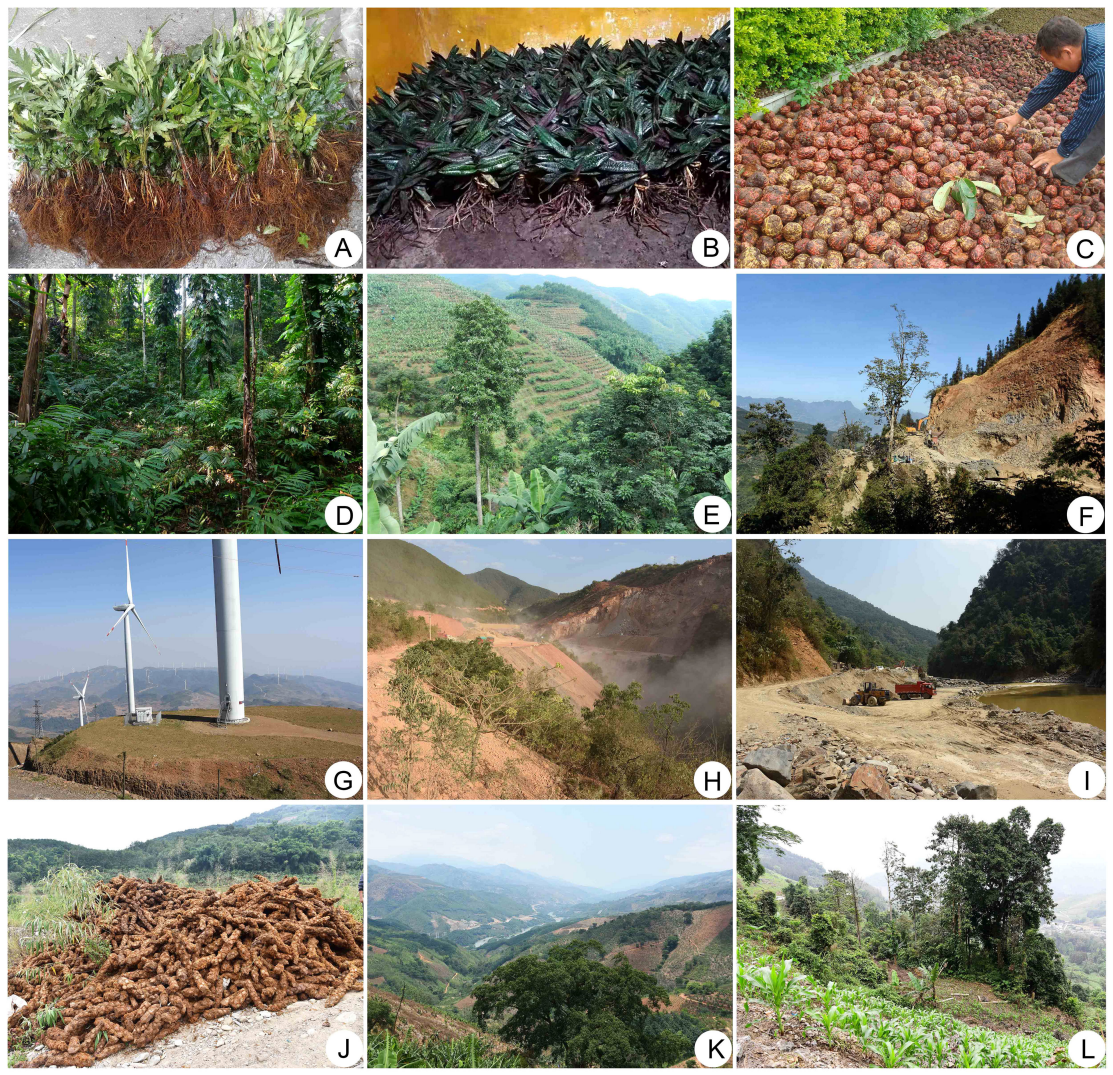
**(A)** Overcollection of *Coptis quinquesecta*, **(B)** Overcollection of *Paphiopedilum* sp, **(C)** Overcollection the fruits of *Manglietia grandis*, **(D)** Planting *Amomum villosum* in *Christensenia aesculifolia* habitat, **(E)** Planting agricultural crops in *Manglietia lucida* habitat, **(F)** Road construction destroyed the habitat of *Hopea chinensis*, **(G)** The construction of wind power generation damaged the habitat of *Rhododendron*, **(H)** Mineral mining destroyed the habitat of *Paphiopedilum wenshanense*, **(I)** Sand mining destroyed the habitat of *Cycas tanqingii*, **(J)** Overcollection of *Cibotium barometz*, **(K)** Reclamation and construction of the Red River Valley, **(L)** Planting crops destroyed the habitat of *Horsfieldia tetratepala*.

The threats to PSESP in Southeast Yunnan that we have investigated and summarized mainly include different types of human interference, limitations imposed on PSESP itself, and natural disasters. Human activity interference mainly includes two aspects, (1) overcollection of resources due to their medicinal, economic, and ornamental value (**Figures 5A–C, J**) and (2) destruction of the natural habitat due to human activities (**Figures 5D–I, K, L**). For the first aspect, there are a few examples as follows. The genus *Paris* has a high medicinal value, which has led almost local residents to dig up when they encounter *Paris* plant. *Coptis quinquesecta* is traditionally used for soaking in wine and medicinal purposes, leading to a shortage of wild resources with less than 100 plants remaining in the natural habitat located on the border between China and Vietnam, constantly facing the danger of extinction (**Figure 5A**). The fruit of *Malania oleifera* is the raw material for extracting nervonic acid and artificially synthesized musk, and the fruits are picked clean every year, resulting in no seedlings in the wild and an inability to naturally regenerate normally. There are also plants with extremely high ornamental value, such as *Paphiopedilum*, *Cycas*, *Camellia*, etc., which are traditionally planted around houses for ornamental purposes. In recent years, due to the large-scale acquisition by illegal traders, they are facing the risk of extinction. For the second aspect, there are also a few examples. The cultivation of economic plants often leads to massive deforestation, such as the planting of industrial tree species like rubber, which has destroyed the natural habitats of *Manglietia lucida* and *Myristica yunnanensis* in Mengla, Jinping, leaving only a few surviving plants on the edges of gullies (**Figures 5E, L**). There are also engineering constructions such as mining, which have caused certain damage to the habitat of *Cycas tanqingii* in the Xiaoheijiang River basin of Lvchun, with trees in the surrounding valleys being cut down and parts of the forest being destroyed, leading to the risk of extinction for the local population (**Figure 5I**). The same situation also occurs in *Hopea chinensis*, *Horsfieldia tetratepala, Paphiopedilum wenshanense* and others.

Except for human interference, the intrinsic limitations of species also play an objective role in the reduction of their populations. The distribution of *Glyptostrobus pensilis* is scattered, leading to strong differentiation between different populations, severe inbreeding, and impeded gene flow. The already limited genetic diversity is further lost, which also exacerbates the endangered status of *G. pensilis* due to low genetic diversity (Wang et al., 2019).

Climate change poses a particular threat to rare and endangered plants with narrow distribution areas, few populations, and special habitats. Once a natural disaster occurs, the remaining population and individuals of this type of PSESP will permanently disappear. At present, the only surviving population of *Christensenia aesculifolia* in the karst area of Hekou County, southeastern Yunnan, is facing sun a risk of extinction (Cai et al., 2019).

## Discussion

### Current conservation status of PSESP in southeast Yunnan

The Southeast Yunnan is a typical limestone region, located on the edge of the northern tropics, preserves a relatively intact tropical mountain ecosystem. It is estimated that the number of higher plant species has exceeded 8,000, making it one of the richest regions in Yunnan in terms of plant species diversity. Despite facing numerous threats, PSESP distributed in southeastern Yunnan has received certain protection actions and scientific research, 96 species (82.76%) have received at least some level of conservation measures, while the remaining 20 species (17.24%) have not received any conservation. 65 species (56.03%) of all PSESP in Southeast Yunnan have received *in situ* conservation, 91 species (78.45%) have received ex situ conservation, 51 species (43.97%) have undergone breeding, and 24 species (20.69%) have received reintroduction, these also demonstrate that Yunnan has provided significant human, financial, and material support in PSESP protection (Yang et al., 2020). This is also consistent with previous research findings: ex situ conservation is one of the most important and effective measures for biodiversity conservation, and at the same time, botanical gardens are one of the most important places for situ conservation and play an important role (Mounce et al., 2017; Chen and Sun, 2018). In addition, the high extinction risk of some PSESP has been reduced through comprehensive conservation actions and scientific research, such as *Craigia yunnanensis*, *Manglietiastrum sinucum*, they have been removed from the latest protection list due to successful rescue conservation (Sun, 2021; Cai et al., 2024a).

Analysis indicates that counties/cities near the China-Vietnam border have higher diversity of PSESP, with Hekou having 44 species, Malipo having 43 species, Maguan having 35 species, and Jinping having 31 species, thus, we should pay more attention to biodiversity conservation in the border areas between China and Vietnam. This is consistent with previous research on PSESP in this region, although there may be differences in quantity (Qian et al., 2020). This distribution pattern is likely related to the lower altitudes, sufficient heat, and abundant rainfall in these areas, creating the characteristic of high species diversity. Although the corresponding nature reserves are distributed in these counties and cities, most species are only found in 1–4 counties/cities, which clearly reflects the narrow distribution area characteristic of PSESP. It is gratifying that the team of Yunnan key laboratory for integrative conservation of PSESP has established two cooperative bases in southeastern Yunnan, and currently more than 80 PSESP have received near situ conservation in the Hekou Botanical Garden for PSESP in Hekou and Laoshan Botanical Garden in Malipo. In addition to the near situ conservation bases, many *in-situ* conservation regions/plots located outside of nature reserves have also been established for species such as *Malania oleifera* (Guangnan County), *Manglietia ovoidea* (Maguan County), *Manglietiastrum sinucum* (Hekou and Jinping Counties) ect. These practical conservation actions will provide strong support for the future PSESP conservation in southeastern Yunnan.

Overall, the PSESP conservation work in southeastern Yunnan has been carried out relatively well, with 96 species have received at least one protective measure and 86 species have conducted at least one scientific research, including 91 species protected through ex situ conservation. At the same time, we should pay more attention to biodiversity conservation in the border areas between China and Vietnam, which have higher PSESP richness.

### Current threatened status of PSESP in southeast Yunnan

Human activities have always been the most significant threat to the maintenance and preservation of plant species diversity. In recent years, with societal development, the distribution information of many species can be easily accessed through the internet, and the convenience of travel has made many previously inaccessible places easily reachable. The rapid development of agriculture, forestry, and road construction has also led to unprecedented destruction of the natural habitats of many plants. Unscrupulous traders, under the guise of business, travel around purchasing PSESP with medicinal, ornamental, and material value at high prices. For profit, many PSESP populations are being destroyed and are constantly at risk of extinction. Due to societal development, humans need to cut down forests and plant economic crops; extensive deforestation has led to the endangerment of many species. At the same time, the construction of various projects also destroys the habitats of plants, and many extremely small wild plant species face severe threats that require timely protection (Cai, 2020).

In this study, a total of 116 PSESP in 21 counties/cities of Southeast Yunnan were counted, among which the family with the highest threat level is Orchidaceae, with a total of 18 species. Overcollection is the main reason why many orchid species are listed as PSESP due to their high ornamental value, such as the famous “slipper orchid” (*Cypipedium* and *Paphiopedilum*), and medicinal value, such as *Dendrobium* (Zhang et al., 2015; Qin et al., 2019; Cai et al., 2024b). At the genus level, the *Cycas* has the most species in the list and also faces various threats, including habitat destruction and plant theft (Xi et al., 2022). This is also consistent with the extensive development and utilization of land in tropical karst areas and the Red River dry hot valley. A total of 81 species are included in the “List of National Key Protected Wild Plants in China” accounting for 69.83%, which is also indicates that the PSESP list and the national key protected plant list have good scientific and consistent development. The destruction of the original habitat is also a factor that cannot be ignored in the high extinction risk faced by PSESP. This issue has been mentioned in many studies of PSESP species, such as road construction, hydropower station construction, and cultivation of new farmland ect, and the destruction of PSESP species is irreversible (Yang et al., 2022b, 2022c; Cai et al., 2024a; Liu et al., 2024; Shen et al., 2024).

For the threats of limitations imposed on PSESP itself and natural disasters, different PSESPs exhibit different characteristics. For the remaining *Christensenia aesculifolia* in southeastern Yunnan, this species can no longer reproduce offspring through spores, whether in the wild or in artificial tissue culture (Cai et al., 2019). *Acer yangbiense*, distributed in Yunnan, not only has low genetic diversity, but also suffers from severe inbreeding and the accumulation of deleterious mutations within the population. The imbalance of sex ratio among mature individuals within the population leads to low fruit set and reproductive difficulties, difficult gene exchange, and similarly faces the danger of extinction (Ma et al., 2022). Since the Quaternary Ice Age, climate change has had a significant impact on the genetic diversity and historical dynamics of animals and plants through species distribution patterns (Ulrey et al., 2016). For example, *Cycas* are suitable for growth in warm and humid ridges, cliffs, and low-altitude river valleys or rock crevices. Climate changes from the ice age to the interglacial period, coupled with the strengthening of the Asian winter monsoon, have scattered *Cycas* in their respective refuges and also hindered gene exchange between populations, leading to gradual differentiation. Currently, *Cycas* in China are facing a severe threat (Fragnière et al., 2015; Liu et al., 2015; Zheng et al., 2016). Irresistible natural factors such as wildfires and landslides can also be causes of plant endangerment (Luo et al., 2018; Xu et al., 2024).

The three most threatened groups of PSESP in southeastern Yunnan are Orchidaceae, Magnoliaceae and Cycadaceae, thus, we should pay more attention to these three groups as their threat status is more severe. Among all 116 PSESP in southeastern Yunnan, there are 81 national key protected plants and 91 severely threatened plants, it indicates that the overall threat status of PSESP in southeastern Yunnan is severe, therefore, conservation and rescue for PSESP are urgently needed.

### Implications for conservation strategies

The introduction and promotion of the concept of PSESP help to draw attention to species that are severely threatened, leading to protective measures for these species. In China, successful conservation measures have been taken for PSESP (Sun et al., 2024). The four conservation measures mentioned above (In situ, Ex situ, Breeding, Reintroduction/Reinforcement) are the most commonly proposed and effective conservation models for PSESP in China. However, due to the complexity of plant species distribution and forest land rights, the *in situ* conservation of many species cannot be well implemented. Many species that are the subject of conservation efforts grow along roadsides, near villages, by rivers, or at the edges of forests, making them susceptible to human activity interference. As a result, *in situ* conservation also faces a severe situation, and it is necessary to increase the promotion of plant protection efforts. In summary, poor protection effectiveness and diverse threats are the current situation faced by PSESP in southeastern Yunnan, and more conservation measures are urgently needed.

In addition to the aforementioned measures, researchers and conservation workers should also focus on the roles of scientific research, germplasm resource conservation, environmental education, and ethnic culture in the protection of extremely small wild plant populations (Cai, 2020). (1) By studying the reproductive biology of PSESP, exploring their reasons for endangerment and weak links in the reproduction process, and taking artificial assistance measures to promote species’ wild fruiting and natural regeneration, this is also a feasible and worthwhile conservation strategy to try (Tang et al., 2020; Yang et al., 2022b). (2) Germplasm resource conservation is also a method to ensure the continuation of species. To prevent PSESP from facing extinction due to man-made or irresistible natural disasters, *in vitro* preservation of germplasm resources is also a feasible and effective method (Lin et al., 2022). (3) Ethnic culture can also play a role in the protection of PSESP. Many species are distributed in the “Fengshui Forests” of ethnic minority areas, and special cultures have led to the better preservation of some endangered PSESP (Liu et al., 2024). (4) Environmental education, including carrying out scientific popularization activities and publicity education activities for the protection of PSESP, can raise public awareness of the protection of PSESP; improving legislation and strengthening publicity on the dangers of destroying PSESP can truly achieve the legal protection of PSESP (Sun et al., 2019a). In the future, the establishment of professional karst botanical gardens or more PSESP ex situ conservation bases in the southeastern Yunnan area should be considered, mainly for the display and ex situ conservation of PSESP and endemic plants in karst areas.

## Data Availability

The datasets presented in this study can be found in online repositories. The names of the repository/repositories and accession number(s) can be found in the article/**Supplementary Material**.
